# Effects of Dietary n-3 LCPUFA Supplementation on the Hippocampus of Aging Female Mice: Impact on Memory, Lipid Raft-Associated Glutamatergic Receptors and Neuroinflammation

**DOI:** 10.3390/ijms23137430

**Published:** 2022-07-04

**Authors:** Lucas Taoro-González, Daniel Pereda, Catalina Valdés-Baizabal, Miriam González-Gómez, José A. Pérez, Fátima Mesa-Herrera, Ana Canerina-Amaro, Herminia Pérez-González, Covadonga Rodríguez, Mario Díaz, Raquel Marin

**Affiliations:** 1 Research Unit, Hospital Universitario de Canarias, 38320 Santa Cruz de Tenerife, Spain; ltaorogo@ull.edu.es; 2 Instituto de Tecnologías Biomédicas de Canarias (ITB), University of La Laguna, 38200 Santa Cruz de Tenerife, Spain; mirgon@ull.edu.es (M.G.-G.); cvarodr@ull.edu.es (C.R.); 3 Laboratory of Cellular Neurobiology, Department of Basic Medical Sciences, Section of Medicine, Faculty of Health Sciences, University of La Laguna, 38200 Santa Cruz de Tenerife, Spain; dperedap@ull.edu.es (D.P.); cvaldesb@ull.edu.es (C.V.-B.); anacanerinaamaro@gmail.com (A.C.-A.); 4 Associate Research Unit ULL-CSIC, Membrane Physiology and Biophysics in Neurodegenerative and Cancer Diseases, University of La Laguna, 38200 Santa Cruz de Tenerife, Spain; 5 Department of Basic Medical Sciences, Faculty of Health Sciences, University of La Laguna, 38200 Santa Cruz de Tenerife, Spain; herperez@ull.edu.es; 6 Instituto de Neurociencia Cognitiva (NeuroCog), University of La Laguna, 38205 San Cristóbal de La Laguna, Spain; 7 Department of Animal Biology, Edaphology and Geology, University of La Laguna, 38200 Santa Cruz de Tenerife, Spain; janperez@ull.edu.es; 8 Laboratory of Membrane Physiology and Biophysics, Department of Animal Biology, Edaphology and Geology, University of La Laguna, 38200 Santa Cruz de Tenerife, Spain; fatimamesaherrera@gmail.com; 9 Department of Physics, Faculty of Sciences, University of La Laguna, 38200 San Cristóbal de La Laguna, Spain; 10 IUETSP (Instituto Universitario de Enfermedades Tropicales y Salud Pública de Canarias), University of La Laguna, 38200 San Cristóbal de La Laguna, Spain

**Keywords:** long-chain polyunsaturated fatty acids supplementation, lipid rafts, aging, diet, hippocampus, neuroinflammation, memory

## Abstract

Long-chain polyunsaturated fatty acids (LCPUFA), essential molecules whose precursors must be dietary supplied, are highly represented in the brain contributing to numerous neuronal processes. Recent findings have demonstrated that LCPUFA are represented in lipid raft microstructures, where they favor molecular interactions of signaling complexes underlying neuronal functionality. During aging, the brain lipid composition changes affecting the lipid rafts’ integrity and protein signaling, which may induce memory detriment. We investigated the effect of a n-3 LCPUFA-enriched diet on the cognitive function of 6- and 15-months-old female mice. Likewise, we explored the impact of dietary n-3 LCPUFAs on hippocampal lipid rafts, and their potential correlation with aging-induced neuroinflammation. Our results demonstrate that n-3 LCPUFA supplementation improves spatial and recognition memory and restores the expression of glutamate and estrogen receptors in the hippocampal lipid rafts of aged mice to similar profiles than young ones. Additionally, the n-3 LCPUFA-enriched diet stabilized the lipid composition of the old mice’s hippocampal lipid rafts to the levels of young ones and reduced the aged-induced neuroinflammatory markers. Hence, we propose that n-3 LCPUFA supplementation leads to beneficial cognitive performance by “rejuvenating” the lipid raft microenvironment that stabilizes the integrity and interactions of memory protein players embedded in these microdomains.

## 1. Introduction

Brain function and lipid homeostasis are progressively compromised during the aging process. Alterations in brain lipid composition have emerged as a key factor contributing to brain aging [1,2,3].

Cholesterol and long chain polyunsaturated fatty acids (LCPUFAs) are amongst the crucial lipid molecules highly represented in the brain. In particular, n-3 (omega-3) LCPUFAs represent a remarkable portion of the physiologic brain lipidome whereas they are poorly produced in the central nervous system. Consequently, LCPUFAs need to be incorporated in the diet, either as n-3 and n-6 polyunsaturated (PUFA) precursors from vegetal sources, such as some grain seeds, or as long chains (20 or more carbon atoms) containing four to six double bonds [4,5,6,7,8,9]. LCPUFAs play a main role in the plasma membrane fluidity and membrane-associated signaling, and can intervene as inflammation mediators [10]. A main n-3 fatty acid in the brain is docosahexaenoic acid (22:6n-3, DHA) that is involved in numerous processes such as neurogenesis, plasticity and neuroprotective functions against neurodegeneration [10,11].

Part of the lipid alterations during physiological aging has been related to lipid raft impairment. Lipid rafts are liquid-ordered domains enriched in cholesterol, sphingolipids and gangliosides such as ganglioside 1 (GM1) [12]. Although less represented, DHA is also present in lipid rafts where it largely determines the physicochemical and biophysical properties of these microstructures, such as lipid–protein and protein–protein interactions [13]. For instance, DHA favors the integration into this hydrophobic environment of numerous proteins that participate in neurogenesis and memory processes [14,15,16]. Remarkably, the loss of lipid raft structure stability may be at the basis of pathological events occurring in Alzheimer’s disease (AD) and Parkinson disease (PD) [17,18].

Lipid rafts are considered functional sites where numerous proteins interact according to stimuli. Raft-related proteins are frequently found in signaling clusters named signalosomes, that are rearranged depending on the extracellular ligand availability. For instance, we have previously characterized the estrogen receptor alpha (ERα) signalosome, formed by a complex set of factors involved in cellular signaling and neuronal survival [19,20,21,22]. Protein components of ER-signalosome includes flotillin and the ionotropic and metabotropic glutamatergic receptors, N-methyl-D-aspartate receptor (NMDAR) and mGluR5, respectively, suggesting the relevance of this signaling platforms in cognitive skills [18,19,23,24,25].

Interestingly, the lipid rafts lipid composition is potentially modifiable by dietary interventions, playing an indirect role in neuronal functioning maintenance in humans [26]. In animal models, dietary n-3 LCPUFA supplementation neutralizes the detriment of avoidance memory and improves the spatial memory in senescence-accelerated prone 8 (SAMP8) mice [27,28,29,30]. Similarly, DHA supplementation restored the contextual fear conditioning memory in aged rats [31].

In parallel with a progressive cognitive decline, enhanced inflammatory activity has been described in the aged brain. Microglia and astrocytes activation has been related with the cognitive impairment associated with much neurological pathology, as demonstrated in animal models of AD [32,33]. These impairments might be related to the aging-induced imbalance between increased proinflammatory and decreased anti-inflammatory factors [34]. Remarkably, the neuroinflammatory processes and proteins involved in lipid raft-mediated signaling are mutually modulated. Noticeably, cytokines may exert distinct patterns of modulation on glutamate receptors; thus, IL-1β modulates differentially the hippocampal expression of the GluN2B subunit of NMDA and the GluA1 subunit of α-amino-3-hydroxy-5-methyl-4-isoxazolepropionic acid (AMPA) receptors, impairing the spatial and recognition memory in rats [35,36,37]. The tumor necrosis factor-alpha (TNF-α), another cardinal pro-inflammatory cytokine, also alters GluA1 AMPA subunit expression in both cultured hippocampal neurons and hippocampal slices [38,39]. In addition, neuroinflammation seems to modulate the physiology of hippocampal mGluR5 [40].

An increasing body of data indicates that LCPUFAs participate in the modulation of neuroinflammatory processes [41], for example, n-3 LCPUFAs increase the expression of anti-inflammatory cytokines, such as IL-10. In parallel, they decrease the expression of pro-inflammatory cytokines, such as IL-1β and TNF-α [42,43] through different strategies, including neutrophils infiltration [44], regulating microglial activation [45] or increasing phagocytosis [46]. Animal studies have shown that supplementation with some DHA derivatives correlates with both a reduced inflammatory response in the brain and a cognitive improvement affected by inflammation [47,48].

The aim of the present study was to investigate the potential effects of a n-3 LCPUFA-enriched diet in cognitive and learning skills in female mice. We also analyzed whether aging progression may alter the lipid composition and functionality of neuronal lipid rafts in the hippocampus, and whether a n-3 LCPUFA diet may revert these age-related alterations. We investigated the potential alterations in hippocampal lipid rafts of the content of glutamatergic receptors such as NMDA and AMPA as well as ERα as part of the molecular mechanisms implicated in aging-mediated hippocampal malfunctioning. Finally, as n-3 and n-6 LCPUFA are precursors for different mediators of inflammation, we investigated the potential inflammatory changes during aging in the hippocampus and the effects of the dietary intervention.

It is worth mentioning that the vast majority of studies in neurosciences, and specifically regarding lipid-diet effects on memory and cognition, have been exclusively conducted in male animals, even though the assumption that females are intrinsically more variable due to the estrous cycle has been disproved and that actually the estrous cycle does not usually need to be considered [49]; therefore, we focused our investigation on female brains as they are more poorly investigated. Indeed, recent reports indicate that the neuroprotective effects of n-3 LCPUFAs differ between male and female animals, related in part to the interactions of estrogens with DHA in the brain [50,51,52].

## 2. Results

### 2.1. A n-3 LCPUFA-Enriched Diet Improves Spatial and Object Recognition Memory Detriment during Aging

Initially, we evaluated the variations of hippocampal-dependent cognitive performance related to aging (6- versus 15-months-old mice) using two different tests: (1) the novel object recognition (NOR) test to analyze the recognition memory ability task; (2) the Barnes maze test to investigate spatial memory [53,54].

**Figure 1:**
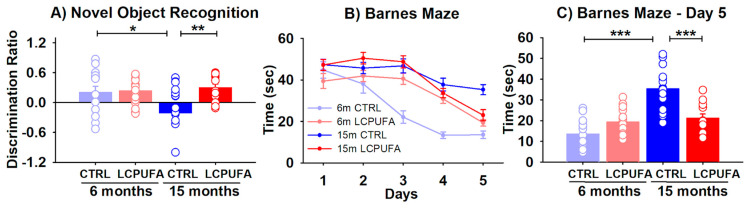
Behavioral tests for the four experimental groups. A n-3 LCPUFA-enriched diet improves spatial and recognition memory in aged female mice. (**A**) Recognition memory was assessed by a novel object recognition task, in which the discrimination ratio was calculated as the difference in exploration time for the novel object minus that for the familiar object divided by the total exploration time. Bars represent the mean ratio ± SEM of 13–18 animals per group. Circles represent each individual measurement. Results were analyzed by a 2-way ANOVA (diet, age) followed by a Tukey’s multiple comparisons test (* *p* < 0.05; ** *p* < 0.01). (**B**) Spatial learning was measured by quantifying the escape time during the 5 days-training. Data were analyzed by a 3-way ANOVA (diet, age, day of training) and individual points in each curve represent the mean ± SEM of 13–16 animals per group. (**C**) Spatial memory achieved in the 5th training day was analyzed separately by representing the mean escape time ± SEM of the four experimental groups, 12–16 animals per group. Results were analyzed by a 2-way ANOVA (diet, age) followed by a Tukey’s multiple comparisons test (*** *p* < 0.001).

Beginning with recognition memory, the analysis of the mice performance in the NOR revealed a significant effect of both diet and an age–diet interaction (*p* < 0.05). The results illustrated in Figure 1 show that aging reduces the ability of female mice to distinguish between a novel object from a previously known one, as illustrated by the reduction of the discrimination ratio of 15-months (−0.21 ± 0.14) with respect to 6-months-old (0.20 ± 0.13) control-fed mice (*p* < 0.05) (Figure 1A). Interestingly, a n-3 LCPUFA-enriched diet reverses this recognition impairment by restoring the discrimination ratio of 15-months-old mice up to 0.30 ± 0.07 (*p* < 0.01).

The spatial learning and memory were assessed at the Barnes maze. During the 5 days-training, all groups learned to escape rapidly out of the platform (training effect *p* < 0.001). Moreover, the statistical analysis of the learning curves showed that both diet and age had a significant effect in the time needed to escape from the platform (*p* < 0.001 and *p* < 0.05 respectively) (Figure 1B).

The escape times from the 5th day of training, when the differences reached a maximum peak, were represented separately in Figure 1C. Here the age, diet and age–diet interaction effects were significant (*p* < 0.05, *p* < 0.001 and *p* < 0.001, respectively). Aging significantly increased the escape time from 14 ± 2 sec in the 6-months-old mice to 35 ± 2 sec in the 15-months-old mice (*p* < 0.001). The n-3 LCPUFA-enriched diet neutralized this memory decline by decreasing the escape time to 21 ± 2 sec in the 15-months-old mice (*p* < 0.001).

The obtained results here show that both the spatial learning and memory and recognition memory are compromised by aging in 15-months-old as compared with 6-months-old female mice. The dietary supplementation with n-3 LCPUFA managed to restore both types of the hippocampal-dependent memory.

### 2.2. A n-3 LCPUFA-Enriched Diet Modulates the Glutamatergic Receptors in Hippocampal Lipid Rafts

We next explored whether the cognitive responses observed above correlate with the expression of glutamatergic receptors involved in learning and memory, particularly AMPA and NMDA receptors [55].

For this purpose, we used samples of lipid raft and non-raft fractions from the hippocampus of the different experimental groups to analyze the expression of selected glutamatergic receptor subtypes by slot blot.

**Figure 2:**
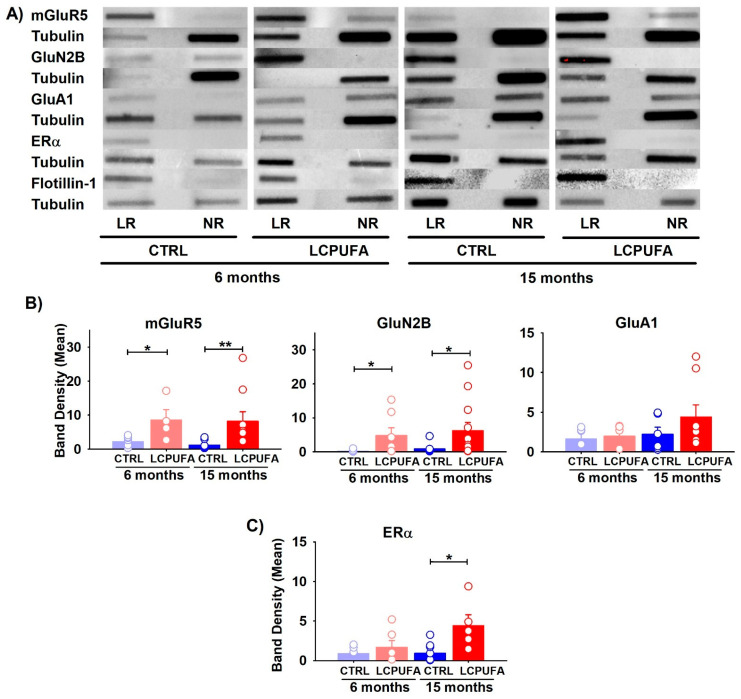
Differential expression of receptors in lipid raft fractions of the hippocampus. (**A**) Representative bands obtained by slot blot technique are shown for each condition. Tubulin expression was used as the charge control and flotillin-1 as the control from lipid raft fractions. In all cases, lipid raft fraction (LR) and non-raft fraction (NR) are shown. (**B**) Effect of age and diet on glutamatergic receptors expression. (**C**) Effect of age and diet on ERα expression. Bars represent the means of band density ± SEM of 4–12 mice per group (bands: mGluR5 6 m *n* = 7 and 15 m *n* = 9; GluN2B 6 m *n* = 8 and 15 m *n* = 12; GluA1 6 m *n* = 5 and 15 m *n* = 8; Erα 6 m *n* = 6 and 15 m *n* = 7). Circles represent each individual measurement. Data were analyzed by a 2-way ANOVA (diet, age) and Tukey’s post hoc test (* *p* < 0.05, ** *p* < 0.01). Data analysis was performed after normalization of the normalization of band intensity relative to tubulin.

To analyze the purity of the lipid raft isolates we used flotillin-1, known to be selectively expressed in these membrane microstructures [56,57]. Our results showed that flotillin-1 was highly expressed in the lipid rafts and completely excluded from the non-raft fractions in the preparations from all groups. Conversely, tubulin, a main component of the submembrane cytoskeleton, was oppositely expressed between the lipid rafts and non-raft membrane fractions (Figure 2A).

We next investigated the contents of the mGluR5, NMDA GluN2B and GluA1 AMPA subunits. With the exception of GluA1, the other glutamatergic receptors were mainly represented in the lipid raft fractions, and only faintly in the non-raft fractions (Figure 2B). Both the mGluR5 and NMDA GluN2B subunit increased in the lipid rafts as a consequence of a n-3 LCPUFA-enriched diet in either the younger or older female mice. For instance, the mGluR5 expression in 6-month-old mice increased four-fold as a consequence of a n-3 LCPUFA supplemented diet, and eight-fold in the 15-month-old mice. Furthermore, the GluN2B expression increased six-fold in the n-3 LCPUFA-fed young animals but did not change in the older animals. In contrast, the GluA1 subunit of the AMPA ionotropic glutamate receptor was unaffected by either a n-3 LCPUFA diet or by age (*p* > 0.05). In agreement, no interaction between the factors was detected for any of these molecules (*p* > 0.05). Graphs of the non-raft fractions can be found in the supplementary material.

These data suggest that the n-3 LCPUFA-enriched diet reverses the effects of aging in the content of both metabotropic and ionotropic receptors, which are crucial to maintain synaptic plasticity in the hippocampus.

### 2.3. ERα Expression Is Enhanced by n-3 LCPUFA in Lipid Rafts of Aged Females

ERα stability has been proven to play a critical role in estrogen protection against cognitive decline during menopause [22]. Here, we analyzed the expression of ERα in hippocampal lipid rafts (Figure 2C). This receptor has been previously shown by our group to modify its trafficking into raft microstructures during female aging, a fact that may affect neuronal survival [58]. In the present study, we found that a n-3 LCPUFA-diet promoted the increase of ERα expression exclusively in aged females. No changes in the expression of this receptor were observed in young animals independently of the diet (*p* > 0.1).

### 2.4. Changes in Lipid Composition of Hippocampal Lipid Rafts with Age Are Partly Reverted by the n-3 LCPUFA-Enriched Diet

Current evidence indicates that a partition of raftophilic proteins within lipid rafts, either as resident or transitorily, is largely dependent on the specific biochemistry of the lipid matrix required for lipid–protein interactions, especially in long-term remodeling. As the glutamatergic markers and membrane ERα displayed an age- and/or diet-dependent association with the lipid raft, we analyzed the composition of fatty acids extracted out from the hippocampal lipid raft fractions from the four experimental conditions.

Assuming the 6-months control animals fed with a commercial diet as representative for standard C57BL/6 mice, we observed a close agreement with the fatty acid data reported previously for hippocampal lipid rafts under control conditions [59,60]. Indeed, lipid rafts contain high amounts of saturates and monoenes, as well as lower PUFA (and LCPUFA) levels, unsaturation index (UI) and peroxidation index (PI) compared to both non-raft fractions and the whole membranes of hippocampal tissue from mice of a similar age [59,60]. Noteworthy hippocampal lipid rafts from 6-months-old animals fed standard or n-3 LCPUFA-enriched diets exhibit similar fatty acids profiles (Table 1); however, some differences were observed regarding both the C18 LCPUFA-precursors and main C20 LCPUFA (arachidonic acid–AA- and eicosapentaenoic acid -EPA), which were lower in the n-3 LCPUFA diet and C22 n-3 LCPUFA (docosahexaenoic acid –DHA- and docosapentaenoic acid -DPA), which displayed higher values in the n-3 LCPUFA diet.

Noticeably, the lipid rafts from older animals fed n-3 LCPUFA-enriched diets retained the fatty profile of young animals, but the animals receiving a standard diet exhibited severe alterations on the specific fatty acids, totals and indexes (Table 1). Thus, comparison between 15-months-old animals receiving the standard or n-3 LCPUFA-enriched diets, revealed a dramatic reduction of DHA (37.3%, *p* < 0.05), total n-3 (35.7%, *p* < 0.05), total PUFA and LCPUFA (around 20%, *p* < 0.05), UI (19.5%, *p* < 0.05) and PI (25.5%, *p* < 0.05), as well as increased total n-6 (+16.9%, *p* < 0.1), and n-6/n3 (+81,3%, *p* < 0.05); therefore revealing a strong effect of diet. Likewise, a significant effect of aging was evident in the animals receiving the standard diet. Indeed, the lipid rafts from 15-months-old mice exhibited a significant depletion of DHA (26. 8%, *p* < 0.05), AA (14.5%, *p* < 0.1), DPA (41.2%, *p* < 0.1), total PUFA and LCPUFA (around 19%, *p* < 0.05), total n-3 (27.9%, *p* < 0.05), total monoenes (17.6%), UI (19.7%, *p* < 0.05) and PI (22.8%, *p* < 0.05). Further two-way ANOVA analyses disclosed that most of these changes exhibited significant interactions between diet and age, with diet generally counteracting the PUFA depleting effect of aging. One important observation in the animals fed the standard diet was that although no significant changes were observed in individual saturates, aging was accompanied by a significant reduction of the PUFA to saturates ratio (PUFA/Sat: 27.5%, *p* < 0.05).

### 2.5. Hippocampal Neuroinflammation during Aging Is Neutralized by a n-3 LCPUFA-Enriched Diet

**Figure 3:**
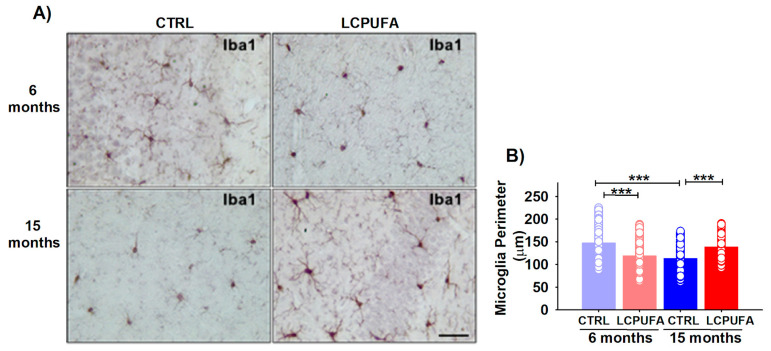
Microglia changes from young and aged female mice. (**A**) Representative images of Iba-1 immunostaining. A n-3 LCPUFA-enriched diet normalizes microglia activation induced by aging in the hippocampus of female mice. Immunohistochemistry was performed in fixed horizontal brain slices from 6- and 15-months-old female mice fed CONTROL or n-3 LCPUFA-enriched diets as indicated in the Methods. Scale bar = 40 μm. (**B**) Microglia perimeter is reduced in the hippocampus of 15-months-old female mice. The n-3 LCPUFA-enriched diet decreases the microglia perimeter in 6-months-old female mice but normalizes it in 15-months-old female mice. Bars represent the mean perimeter length (micrometers) ± SEM from 188–314 microglial cells per group, measured as described in the Methods. Circles represent each individual measurement. Data were analyzed by a 2-way ANOVA (diet, age) and Sidak’s post hoc test (*** *p* < 0.001). *n* = 4 animals per group.

As previously mentioned, neuroinflammation is characterized by two remarkable phenomena: microglia activation and astrocytic proliferation and hypertrophy. When activated, microglial cells take an amoeboid-like morphology, reducing their cell perimeter and ramification [62]. Iba-1 is considered a macrophages’ marker that is expressed specifically in microglial cells in the brain [63]; therefore, to analyze the microglia activation in the hippocampus of young and aged female mice, and the potential modulatory effect of n-3 LCPUFA supplementation, we analyzed the microglial cells’ perimeters in paraffin-embedded sections by Iba-1 immunohistochemistry (Figure 3A).

Our results show a significant effect of both age and an age–diet interaction in the hippocampal microglia perimeter (*p* < 0.001). Aging activated the hippocampal microglia, decreasing the cell perimeter from 147.0 ± 2.8 μm in 6-months-old mice to 112.8 ± 1.7 μm in 15-months-old female mice (*p* < 0.001). A n-3 LCPUFA-enriched diet normalized the microglia activation in the hippocampus of 15-months-old female mice, increasing its perimeter to 138.0 ± 2.1 μm (*p* < 0.001). In comparison, the n-3 LCPUFA-enriched diet activated the hippocampal microglia in 6-months-old female mice, reducing its perimeter to 118.4 ± 2.4 μm (*p* < 0.001) (Figure 3B).

Astrocytes also contribute to the innate immune response into the central nervous system [64], and astrocytic proliferation and hypertrophy have been observed in many neuroinflammatory processes, including aging-related pathologies [32]. Consequently, we also studied the hippocampal astrocytes activation in young and aged female mice by quantifying its covering area in GFAP-stained paraffin-embedded sections. GFAP constitutes intermediate filaments which specifically organize the cytoskeleton of astrocytes [65].

**Figure 4:**
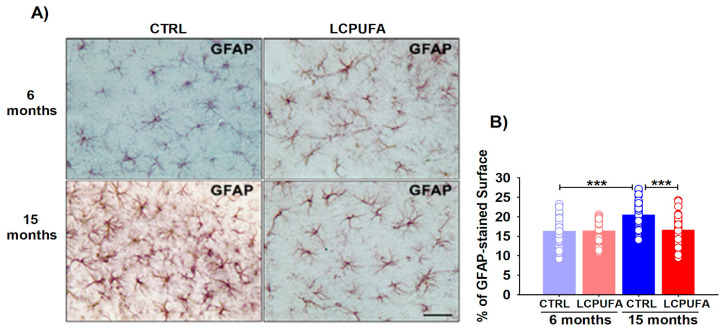
Astrocytic changes from young and aged female mice. (**A**) Representative images of GFAP immunostaining. A n-3 LCPUFA-enriched diet normalizes astrocytes hypertrophy induced by aging in the hippocampus of female mice. Immunohistochemistry was performed in fixed horizontal brain slices from 6- and 15-months-old female mice fed CONTROL or n-3 LCPUFA-enriched diets as indicated in the Methods. Scale bar = 40 μm. (**B**) Astrocytes covering area is increased in the hippocampus of 15-months-old female mice. A n-3 LCPUFA-enriched diet normalizes the astrocyte’s covering area. Bars represent the mean astrocytes covering area (%) ± SEM from 33–41 40× fields per group, measured as described in the Methods. Circles represent each individual measurement. Data were analyzed by a 2-way ANOVA (diet, age) and Tukey’s post hoc test (*** *p* < 0.001). *n* = 4 animals per group.

Both age, diet and the age–diet interaction significantly affected the astrocytes covered in the mice hippocampi (*p* < 0.001, *p* < 0.05 and *p* < 0.05, respectively). Astrocytes from the 15-months-old female mice were hypertrophied as compared with the 6-months-old ones (covering an area of 20.4 ± 0.6% vs. 16.2 ± 0.7%, respectively, *p* < 0.001). The n-3 LCPUFA-enriched diet reduced the astrocyte activation, restoring its covering area in the hippocampi of the 15-months-old female mice to (16.5 ± 0.6%, *p* < 0.001); hence, similar to the values in the other three groups (Figure 4B). The n-3 LCPUFA effects on the astrocyte phenotype were only observed in aged females, whereas no significant differences were detected in young animals independently of the diet (Figure 4A).

In summary, these results indicate that aging triggers a neuroinflammatory process in the hippocampus of female mice. The microglia are activated and astrocytes are hypertrophied in the hippocampus of 15-months-old in comparison with 6-months-old female mice. The n-3 LCPUFA-enriched diet significantly reversed the neuroinflammatory progression, turning back both the astrocytes and microglia to a young state.

### 2.6. Hippocampal Content of Proinflammatory Cytokines Is Modulated by Aging and an n-3 LCPUFA-Enriched Diet

Both astrocytes and microglia release a plethora of chemical mediators (e.g., cytokines, chemokines, etc.), that trigger inflammatory and/or anti-inflammatory pathways. Amongst the pro-inflammatory cytokines, IL-1β and TNF-α play central roles in neuroinflammation as they are released by astrocytes and activated microglia [66,67,68]. We analyzed both the IL-1β and TNF-α hippocampal levels by ELISA.

**Figure 5:**
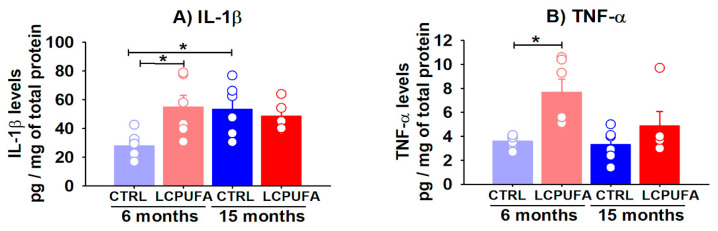
Hippocampal levels of IL-1β and TNF-α are differentially modulated by age and an n-3 LCPUFA-enriched diet. Hippocampal levels of IL-1β and TNF-α were analyzed by ELISA in young (3–6 months-old) and old (12–15 months-old) female mice. (**A**) IL-1β levels are increased in the hippocampus of aged mice. The n-3 LCPUFA-enriched diet increased the IL-1β levels in the hippocampus of young mice. (**B**) Hippocampal TNF-α levels are not modulated by age but the n-3 LCPUFA-enriched diet induced a significant increasing global effect on them. Values are expressed as pg of IL-1β or TNF-α by mg of total protein in the samples, and bars represent the mean ± SEM of 5–7 mice per group. Circles represent each individual measurement. Data were analyzed by a 2-way ANOVA (diet, age) and Tukey’s post hoc test (* *p* < 0.05).

Concerning the IL-1β, a two-way ANOVA revealed no significant global effects for diet or age as the main factors, but interestingly the interaction of age–diet was found to be significant (*p* < 0.05), which indicates a complex regulation by n-3 LCPUFA of IL-1β production depending on the changes associated with aging. Our data show that IL-1β levels increased by 92.1% in the hippocampus of 12–15 months-old mice (53.4 ± 7.4 pg/mg protein) with respect to 3–6 months-old mice fed a standard diet (27.8 ± 3.3 pg/mg protein). On the other hand, a n-3 LCPUFA-enriched diet significantly increased the IL-1β levels by 96.8% in the hippocampus of 3–6 months-old mice (from 27.8 ± 3.3 to 54.7 ± 8.3 pg/mg protein (Figure 5A).

Regarding the TNF-α hippocampal levels (Figure 5B), a significant global effect of a diet devoid of interaction with age was found by a two-way ANOVA test (*p* < 0.01). The n-3 LCPUFA-enriched diet significantly increased the hippocampal TNF-α levels by 113.9% in the 3–6 months-old mice (from 3.6 ± 0.2 to 7.7 ± 1.1 pg/mg protein, *p* < 0.05) and tended to increase them in the 12–15 months-old mice (from 3.3 ± 0.5 to 4.9 ± 1.2 pg/mg protein).

## 3. Discussion

Cognitive and activity decline with aging have shown acute sex differences in several behavioral tasks and activity measurements [69,70,71,72]. Dietary supplementation with n-3 PUFA has shown to improve age-related cognitive decline and neurodegeneration, particularly in the hippocampus [73,74,75,76]; however, only a few studies have reported the potential benefits of PUFA dietary interventions in the female brain during aging. In the present work, we have found that dietary supplementation with n-3 LCPUFAs can counteract aged-induced memory impairments in female mice.

We observed an increase in the spatial learning ability of old females fed with the n-3 LCPUFA-enriched diet, as reflected by lower escape times in the Barnes maze test. Our results are in line with a previous report in which a n-3 LCPUFAs-enriched diet improved spatial memory in a Morris water maze in 21-months-old male mice [75]. Likewise, in accelerated senescence models of aging (SAMP8 mice), a diet enriched with DHA-containing phospholipids managed to improve spatial memory in the Barnes maze [77]. Similarly, a DHA + EPA-enriched diet had similar memory improvement in ApoE4 transgenic mice, slightly defined as a model of AD [78]; however, in these studies, no gender differences were established. Regarding female mice, a DHA-enriched diet improved the spatial memory of 17-months-old female Kunming mice in a passageway water maze test [79]. Moreover, a less-specific fish oil-enriched diet restored spatial memory in 10-months-old female OF1 mice in a Morris water maze [80]. Altogether, these data are in line with our results, concluding that dietary n-3 LCPUFA enrichment can improve spatial memory performance in aged mice.

Compared with young female mice, older ones showed difficulties remembering a previously explored object when presented together with a new one. In contrast, when fed with the n-3 LCPUFA-enriched diet, the discrimination index of the older mice was similar to the values in the younger mice. To our knowledge, this is the first demonstration that a n-3 LCPUFA-enriched diet can counteract recognition memory alterations induced by aging in female mice. In correlation with our results, previous data in aged C57 male mice have shown that a n-3 LCPUFA diet improved recognition memory [75]. Other studies using AD-like mice strains, such as Tg2576 mice and ApoE4 transgenic mice, have previously demonstrated that a DHA-enriched diet improves the novel object recognition related to aging [78,81].

Our results on hippocampal memory tests indicate that spatial learning and recognition memory were impaired by aging and restored by dietary supplementation. In support of our observations, previous reports have shown that LCPUFA-selected diets can reduce the long-term potentiation (LTP) impairment associated with aging in rats; thus, aged animals fed with a combination of different PUFA classes resulted in an improvement of LTP-deprived activity similar to the levels of young rats [82]. Different glutamate receptors are involved in the efficiency of synaptic transmission and plasticity in LTP. For instance, some forms of hippocampal LTP are mediated by NMDA receptors [83]. In addition, although less characterized, mGluRs also participate in the processing of spatial memory in the hippocampus [84,85,86]. In this sense, it has been described as an increase in mGluR5 expression followed by an LTP induction at the perforant path–dentate gyrus synapse [87], and that blockade of this receptor leads to a deficit in the working and reference memory. In addition, mGluR5 is implicated in hippocampal LTD, a process related to cognitive flexibility, memory extinction and reversal learning [88].

Therefore, we next investigated whether changes in the hippocampal lipid rafts may be involved in the detriment of memory test performance in older females. Lipid rafts are known to be signaling platforms for glutamate receptors related to cognitive skills [89,90,91]. For instance, some data have suggested that lipid raft-aging is affected by gender and may be prevented by improving the balance of LCPUFA in these membrane microstructures [92]. Here, considering the hippocampal memory preservation in aged female mice following a n-3 LCPUFA-enriched diet, we explored whether this phenomenon may be related to a preservation of the lipid raft integrity that may stabilize glutamatergic receptors improving the plasticity processes.

We studied whether the potential changes in trafficking and dynamics of both the ionotropic and metabotropic glutamatergic receptors may correlate with hippocampal lipid raft integrity and memory and learning skills. NMDA receptors, specifically the GluN2B subunit, were found to be increased specifically in lipid raft fractions following a n-3 LCPUFA diet in both groups of age. Importantly, compared with the aged animals fed a standard diet, the aged female mice receiving n-3 LCPUFA showed a higher content of GluN2B in lipid rafts correlating with the better spatial learning observed. Similar findings were found in the expression of mGluR5 receptors that was enhanced in the aged mice following the n-3 LCPUFA diet as compared to the control mice. Although we cannot establish a cellular mechanism underlying this finding, it is enticing to hypothesize that preservation of the association of these glutamatergic receptors with the lipid raft by a n-3 LCPUFA diet may result in improved synaptic plasticity mechanisms, such as LTP and LTD during brain aging. In support of this, it has been previously reported that deficiencies in n-3 LCPUFA such as DHA in the brain, correlates with a reduction in the GluN2B amount leading to LTP impairment [93]. Moreover, the hippocampus of rats under treatment with DHA increases the expression of the NMDAR subunit GluN2B as well as in DHA-enriched cultured mouse hippocampal neurons [94,95].

In our experimental hippocampal lipid raft samples, we also tested the potential effects of n-3 LCPUFA diet in the dynamic of ERα during aging progression. This receptor is embedded in signalosomes, where it exerts neuroprotective actions by contributing to membrane lipid homeostasis in conjunction with DHA (reviewed in Ref. [22]). It is known that the ERα content is decreased in neuronal lipid rafts during female brain aging, a fact that correlates with a higher risk of neurodegeneration [58]. Noticeable, transgenic ERαKO mice lacking ERα expression in the brain show an impaired memory and a detrimental hippocampal cognitive function [96]. Therefore, we wondered whether the presence of this receptor in lipid rafts may be modulated by the n-3 LCPUFA diet used in this work. We found that ERα is highly expressed in the hippocampal lipid rafts of animals treated with a lipid diet versus age-matched controls receiving the standard diet. This may contribute to the LTP balance in the hippocampus as previously suggested [83], and may support the enhancement of memory and learning in aged females fed with a n-3 LCPUFA-enriched diet.

Numerous pieces of evidence have demonstrated that the lipid matrix is a critical determinant of membrane proteins’ functionality. Further, it is known that the assembly of stable membrane multimolecular clusters and membrane protein partitioning depends on specific bilayer physicochemical properties, in particular in membrane microdomains, including lipid rafts [97,98,99]. Hence, we explored the potential relationship between the lipid raft lipid matrix in the redistribution of glutamate receptors and subunits observed here. The data revealed that lipid rafts undergo a series of changes which collectively fit the “lipid raft aging” hypothesis described previously for murine models and human brains [17,92]. Hence, the hippocampal lipid rafts from the 15-months-old mice fed the standard diet exhibited a depletion of the main 20-carbons n-3 and n-6 LCPUFA (EPA and AA), total monoenes (mainly n-9 isomers), as well as the unsaturation and peroxidability indexes. These changes associated with aging were accompanied by a significant reduction in the PUFA to saturated fatty acids ratio, which suggests a physical hindrance to the lateral movements in aged animals. Interestingly, administration of the n-3 LCPUFA-supplemented diet abolished the aging effects and restored the lipid matrix to values found in young littermates. Altogether, these results suggest that aging increases the steady-state membrane order and lipid raft microviscosity as demonstrated previously [60]. Moreover, administration of a n-3 LCPUFA-enriched diet reverted most changes associated with aging, therefore contributing to lipid raft stability and homeostasis throughout the lifespan. Although the receptor trafficking and domain distribution depends on different regulatory factors, it is clear that the higher microviscosity in the hippocampal lipid rafts from older animals would impose a limitation for the lateral movements needed for receptor clustering in the lipid rafts, presumably impairing the glutamate signaling required in cognitive processes [75,77,78,79,81,100]. The finding that the n-3 LCPUFA-containing diet restores lipid rafts’ lipid neurochemistry strongly agrees with multiple studies demonstrating cognitive improvements upon n-3 LCPUFA supplementation [26,27,73,75].

Neuroinflammation is considered another main feature of brain aging and neurodegeneration. Neuroinflammation is observed to increase in the aged hippocampus where it contributes to cognitive impairment related to both normal and pathological aging [34,101]. Previous studies have reported the anti-inflammatory effects of n-3 LCPUFA in different experimental models [41,102], although data are missing related to the potential anti-inflammatory effects of n-3 LCPUFA dietary interventions in aged females. It is plausible that the neuroinflammatory response may be different in female mice. For instance, significant sex-based differences have been reported in the profile and content of microglia-expressed cytokines at early postnatal stages [103] as well as in adult and aged animals [104,105]. Likewise, it is known that the activation of estrogen receptors leads to protection against neuroinflammation [106]. These data support that there may be a sexual dimorphism in the neuroinflammation response during aging that has not yet been explored. Here, we wondered if the n-3 LCPUFA-mediated recovery of cognitive function we observed in aged female mice could be related to a mitigation of aging-induced neuroinflammation.

Firstly, our data showed an increase in the neuroinflammatory response in older female mice when compared with younger ones. Specifically, we observed an enhancement in both the microglia and astrocytes activation in the hippocampus of 15-months-old compared with 6-months-old mice. These observations are in line with previous studies in which not only aging-mediated activation and morphological changes in hippocampal microglia and astrocytes have been demonstrated, but also phenotypic and gene expression alterations [101]. Interestingly, in the aged mice group fed with the n-3 LCPUFA diet, we observed a normalization of the hippocampal microglia and astrocytes activation as compared to age-matched controls fed with the standard diet. These data suggest that n-3 LCPUFA uptake in the brain may trigger immune cell patterns to revert the inflammatory processes mediated by microglia and astrocytes in the hippocampus associated with cognitive benefits. To our knowledge, this is the first demonstration of n-3 LCPUFA modulation of hippocampal inflammatory features in aged female mice in the absence of other concomitant pathologies. Other previous works conducted in male rodent models support our data. For instance, it has been previously shown that a combination of DHA and EPA supplementation decreased microglia markers and restored astrocytes morphology in the hippocampus of 22-months-old C57 male mice. These cellular changes were associated with the amelioration of spatial and recognition memory skills in these animals [73]. Hippocampal astrogliosis was also reduced by a LCPUFA combination of DHA, EPA and n-3 docosapentaenoic acid (DPA) supplementation in aged male mice with forebrain cholinergic depletion, in correlation with recognition memory restoration [107]. The beneficial effect of n-3 LCPUFA in reverting the inflammatory scenario appears to be also achieved with specific individual n-3 LCPUFA species; thus, in a rat neuroinflammation model induced by central injection of IL-1β, EPA dietary supplementation ameliorated microglia and astrocytic activation in the hippocampus [108]. Additionally, DHA supplementation decreased the microglial markers in the hippocampus and prevented contextual fear conditioning impairments in aged male rats fed with a processed food diet [31].

The fact that a n-3 LCPUFA diet modulated the astrocytic and microglia activity in correlation with hippocampal functioning restoration during aging led us to explore the potential mechanisms involved. It has been previously characterized that glia-released mediators such as chemokines and cytokines contribute to the functional deterioration of aged brains [34,101,109,110,111,112]. Both cytokines have been repeatedly demonstrated to increase in the aged hippocampus [101]. We observed that levels of IL-1β were increased in the hippocampus of either 3–6 months-old mice fed with n-3 LCPUFA or 12–15 months-old female mice, a fact that abolished the effect of diet alone. In contrast, TNF-α was enhanced in the young animals as a result of the n-3 LCPUFA diet, but not in the aged mice. These data correlate with the observed microglial activation in these mice. Furthermore, our data showed that the n-3 LCPUFA-enriched diet did not modify the hippocampal levels of either IL-1β or TNF-α in aged female mice. This is in contrast with previous studies in aged male mice where a n-3 enriched diet reduced the hippocampal expression of TNF-α but not IL-1β [73]. In vitro assays have reported that n-3 LCPUFA are precursors in the prevention of neuroinflammation [113]. DHA and EPA reduce the expression of some proinflammatory markers such as TNF-α, interleukin-6 (IL-6), nitric oxide synthase (NOS) and cyclo-oxygenase-2 (COX-2) in stimulated BV-2 cultured microglia [114]. These changes may take place through microglial inactivation. In support of this, an intravenous injection of DHA phospholipids reduced IL-6 and IL-1β hippocampal expression in LPS-challenged C57 mice [115]. The fact that IL-1β and TNF-α remained invariable following a n-3 LCPUFA treatment suggests that in healthy aged female hippocampi, distinct proinflammatory pathways may induce specific cytokine expression. We believe that the activation of astrocytes and microglia to induce different cytokines may be determined by several parameters such as a n-3 LCPUFA diet and duration, gender, etc. Moreover, the phenotypes of activated astrocytes or microglia are pretty much diverse to that which they were thought to be. Thus, in each particular situation, and in our case the aged female hippocampus without any other pathology, some cytokines and no others may be released; therefore, further studies on other neuroinflammatory markers (e.g., NOS, COX-2, IL-6, etc.) will be needed to elucidate the precise mechanism by which glial modulation by our n-3 LCPUFA-enriched diet contributes to cognitive restoration.

It is worth mentioning some interesting observations obtained in 6-months-old mice with the n-3 LCPUFA-enriched diet (unpublished data). Mice from this experimental group needed more time to find the escape from the Barnes maze in the 3rd and 4th days of training, behaving similarly to young mice. In parallel, we observed a significant increase in the activity of microglia in young mice fed with n-3 LCPUFA as compared to the control-diet mice. Interestingly, this activation was in parallel with increased hippocampal levels in IL-1β and TNF-α (data not shown). This trend was not observed in the protein or lipid markers of the hippocampal lipid rafts. Additional experiments may be performed in order to further explore the mechanisms involved in this phenomenon.

To our knowledge, these n-3 supplementation-mediated effects on the spatial learning and neuroinflammation of young mice have not been described before in the few studies that have conducted similar comparisons [73,116] except one, in which a slight trend was observed in the 5–6th training day in a Morris water maze in n-3-supplemented 6-months-old wild type animals [117]. Nevertheless, again, all these studies have been conducted in male mice; therefore, it would be plausible that previously mentioned sex-based differences in both brain function and neuroinflammatory processes make young females also more susceptible than males to imbalances in brain lipids.

## 4. Materials and Methods

### 4.1. Animals

**Scheme 1:**
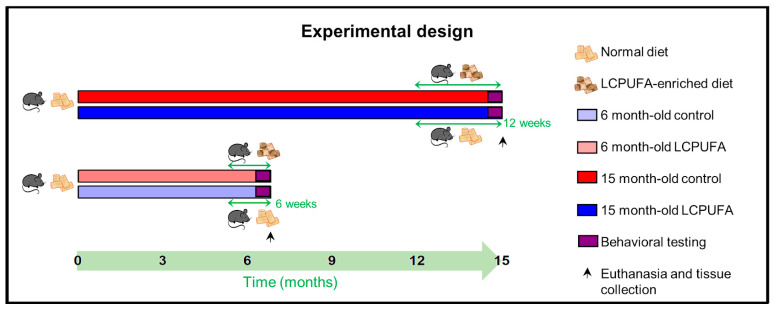
Experimental design. The chronogram shows a summary of the experimental procedures undertaken by the different age groups. Briefly, the 6-month-old LCPUFA group was changed to the LCPUFA-enriched diet 6 weeks before culling and tissue collection, whereas the 15-month-old LCPUFA group were changed 12 weeks before. The behavioral test battery was performed the two weeks prior to culling in all groups targeted. *n* = 40 animals per group.

Four different experimental cohorts of C57BL/6 littermate mice were established: 6-month-old females fed with a control diet (6 m Ctrl), 6-month-old females fed with a n-3 LCPUFA-enriched diet (6 m LCPUFA), 15-month-old females fed with a control diet (15 m Ctrl) and 15-month-old females fed with a n-3 LCPUFA-enriched diet (15 m LCPUFA) (see Scheme 1). The cohorts were housed together and maintained under standard animal house conditions, a 12 h light–dark cycle and ad libitum food and water. All procedures involving the ≈160 mice used in this study were performed in accordance with local and national guidelines (European Council Directive 86/602/EEC) and had been approved by the institutional Research Ethics and Animal Welfare Committee (CEIBA 2019-0346).

### 4.2. Nutritional Enrichment

The control-diet groups were fed with a standard Teklad Global 14% Protein Rodent Maintenance Diet by Envigo ad libitum. The 6- and 15-months-old n-3 LCPUFA-enriched diet groups were also fed ad libitum with this diet up to 6 and 12 weeks before culling, respectively. Then, they were supplemented with Eupoly-3 DHA oil by Biosearch Life, containing a 2.1:1 EPA-to-DHA ratio enrichment, custom added to the standard diet during manufacturing by the supplier, for the rest of the experimental period. The detailed lipid content of both the standard and N-3 LCPUFA-enriched diets is provided in Table 2.

### 4.3. Behavioral Tests

A comprehensive behavioral test battery was designed and implemented to characterize the cognitive phenotype of the four experimental cohorts as previously described [70]. Behavior testing was carried out always during the light phase of the cycle. Every trial of every test was recorded and complied, and the video files were batch analyzed post facto by a technician blind to age and the diet fed.

#### 4.3.1. Novel Object Recognition

A continual trial arena was set up to evaluate the spontaneous object recognition (SOR) memory in our cohorts [118]. A four-day habituation period was allowed, during which the mice were progressively exposed to both areas of the apparatus and trained to shuttle between them. Two identical copies of the same object were placed in the object area and the mouse was allowed to interact with them for 2 min (exploration phase), after which the animal shuttled back to the holding area for 1 min, while one of the copies of the object was replaced for a different one. Then, the mouse was shuttled to the object area, where it was allowed to interact with both objects for 2 more minutes (test phase). The position of the new object was changed between the mice in a semi-random sequence. The object discrimination (D2) in each test phase was measured by calculating the difference in exploration time for the novel object minus that for the familiar object divided by the total exploration time [119].

#### 4.3.2. Barnes Maze

A Barnes maze was set up to evaluate the spatial memory and learning in our cohorts [120,121]. In every trial, the mice were carefully placed in the middle of the arena facing a different direction in a random sequence, allowing exploring for 1 min. Every mouse was subjected to a 15-trial block scheduled as 3 experiments a day, separated by 30 min, over 5 consecutive days. The trials were recorded and the time spent on the arena before finding the hidden box (escape time) was quantified on every trial. The mean of the three daily trials of each animal was considered for the statistical analysis.

### 4.4. Lipid Raft Extraction from Total Hippocampal Lysate

Mice fed with the control and n-3 LCPUFA-enriched diets were sacrificed by cervical dislocation and both hippocampi were rapidly dissected, immediately frozen in liquid nitrogen and maintained at −80 °C until processing. The hippocampal tissue was homogenized with a polytron (Kinematica, Switzerland) in 1 mL of ice-cold buffer A (150 mM NaCl, 10 mM MgCl2, 50 mM Tris/HCl pH = 8.0) with 1% Triton X-100, 5% glycerol and 5 mM β-mercaptoethanol, and supplemented with proteases inhibitors (1 mM PMSF and complete cocktail) and phosphatases inhibitors (20 mM NaF, 1 mM Na_3_VO_4_). The procedures were performed as previously described [122]. Briefly, the hippocampus homogenates were processed in a sucrose gradient and ultracentrifuged (at 150,000× *g* for 18 h at 4 °C in a Beckman (Brea, CA, USA) SW41Ti rotor). Twelve 1 mL fractions were carefully collected. The lipid rafts were extracted between fractions 2 and 3. The 13th fraction (non-raft fraction) was obtained from the reconstitution and sonication in buffer A.

### 4.5. Slot-Blot Analysis

A 200 ng amount of total protein from each gradient fraction was spotted onto a nitrocellulose membrane sealed on a slot-blot set-up (Bio-Rad, Hercules, CA, USA). The membranes were blocked with every blot blocking buffer (Bio-Rad) for 5 min at RT and incubated as follows. The immunodetection of flotillin-1, estrogen receptor alpha (ERα), mGluR5, GluN2B and GluA1, the subunits of NMDA and AMPA receptors, respectively, was performed by incubation with specific antibodies (Anti-Flotillin 1, 1:1000, ab41927; Anti-ERα 1:200, sc-542; Anti-Metabotropic Glutamate Receptor 5, 1:1000, ab76316; Anti-GluN2B, 1:1000, ab65783; and anti-GluA1, 1:1000, ab109450) overnight at 4 °C, followed by incubation with the corresponding secondary-HRP antibody. All antibodies were purchased from Abcam (Cambridge, UK), except the anti-ERα antibody, which was purchased from Santa Cruz Biotechnology (Dallas, TX, USA). In all cases, the signal was developed with a ClarityTMWestern ECL Substrate (Bio-Rad). The detection was performed with a Chemie-Doc MP Imaging System (Bio-Rad), and its optical density analyzed using the Image Lab software. The bands intensity was always normalized referring to α-tubulin bands intensity as the protein loading control.

### 4.6. Lipid Analyses

Total lipids from the lipid raft fractions from the hippocampus homogenates were extracted with chloroform/methanol (2:1 *v*/*v*) containing butylated hydroxytoluene (0.01%) as the antioxidant, according to Folch et al. (1957) [123] with small modifications. The organic solvent was evaporated under a stream of nitrogen, and the lipid content determined gravimetrically and stored in fresh solvent at −20 °C until further analysis. Lipids from the lipid rafts were subjected to acid-catalyzed transmethylation using 1 mL of toluene and 2 mL of 1% sulfuric acid (*v*/*v*) in methanol for 17 h in a heater at 50 °C. The resultant fatty acid methyl esters (FAMEs) and dimethylacetals (DMAs) were purified by thin layer chromatography (TLC), and quantified using a TRACE GC Ultra (Thermo Fisher Scientific, Waltham, MA, USA) gas chromatograph equipped with a flame ionization detector, with helium as the carrier gas. Individual FAME and DMA peaks were identified by referring to authentic standards (Mix C4-C24 and PUFA n° 3 from menhaden oil (Supelco Inc., Bellefonte, PA, USA).

### 4.7. Immunohistochemistry

Four female mice from each experimental group were intracardially perfused with saline followed by 4% paraformaldehyde (PFA) in a 1 M phosphate buffer (PB) to fix their brains under terminal anesthesia with 160 mg/kg chloral hydrate. After overnight PFA, the brains were dehydrated through successive higher grading ethanol solutions and xylene and embedded in paraffin. Horizontal 10 μm-thick sections were obtained on a microtome (Shandon Finesse 325; Thermo Electron Corporation, Waltham, MA, USA). For the immunohistochemical procedure, after being deparaffinized and rehydrated, the sections were washed twice in 50 mM Tris buffered saline (TBS) pH 7.6 (50 mM Trizma Base, 0.9% NaCl). The sections were incubated overnight in a humidified chamber at room temperature with the primary antibodies prepared in TBS: rabbit polyclonal anti-ionized calcium-binding adapter molecule 1 (Iba-1) (1/200, #019-19741, Wako, Zaragoza, Spain) and rabbit polyclonal anti-glial fibrillary acidic protein (GFAP) (ready to use, #IR524, Agilent Dako, Santa Clara, CA, USA). Then, the sections were washed thrice in TBS and incubated for 1 h with biotinylated secondary goat anti-rabbit antibody (1/200, #OS03B, Sigma-Aldrich, Saint Louis, MO, USA). After several washes in TBS, the sections were incubated for 1 h with streptavidin-biotin peroxidase complex (1/200, #189730, #203194, Calbiochem, Darmstadt, Germany) and developed using a TBS solution containing 0.04% 3,3′-diaminobenzidine (DAB), 0.04% ammonium nickel (II) sulfate, and 0.01% hydrogen peroxide by a 5 min immersion. Afterwards, the sections were dehydrated, cleared in xylene and cover-slipped with Eukitt (O. Kindler, Freiburg, Germany).

### 4.8. Microglia and Astrocytes Analysis

Fields randomly selected from 5 sections containing the dorsal hippocampus of 4 animals per group were photographed using a Leica DM 1000 LED microscope (Leica, Wetzlar, Germany). To study the microglia activation, we measured the perimeter of Iba-1-positive microglial cells. To measure the astrocytes hypertrophy we quantified the percentage of the total field area occupied by GFAP-positive astrocytes. The images were analyzed by ImageJ (National Institutes of Health, Bethesda, MD, USA). Following calibration, the microglial cells’ perimeters were measured using the *Count and measure objects* function selecting the Iba-1-stained cells manually and Perimeter as the chosen parameter. The perimeter of 188–314 microglial cells was analyzed per group. To quantify the area covered by astrocytes, the format of the images was converted to 8-bit. The Auto Threshold function was used in order to apply intensity thresholds to distinguish between the GFAP-stained areas and the background color. Masks from the images were checked individually to ensure that the area selected was equivalent to the GFAP staining. The GFAP-stained area was analyzed in 33–41 fields per group.

### 4.9. ELISA Immunoassay

The 3- and 6-months-old female mice and 12- and 15-months-old mice were analyzed together since no significant differences were observed. The mice were sacrificed by cervical dislocation and both hippocampi were rapidly dissected and immediately frozen in liquid nitrogen and maintained at −80 °C until processing. The hippocampal tissue was homogenized with a polytron (Kinematica, Switzerland) in a cold homogenizing buffer (66 mM Tris/HCl pH = 7.4; 35 mM NaCl; 1% SDS, 1 mM EGTA, 5 mM EDTA, 10% glycerol, 1 mM NaF, 1 mM Na_3_VO_4_; 1 mM MPSF and 1× Complete Protease Inhibitor Cocktail). The samples were centrifuged at 13.000× *g* and 4 °C for 20 min, after which the supernatants were collected and stored at −20 °C until used. The total protein concentration was measured by a bicinchoninic acid (BCA) assay (BCA, Sigma-Aldrich/Merck, Darmstadt, Germany). The hippocampal total extract samples were used to measure the tissular levels of proinflammatory cytokines by ELISA. Commercial kits for the IL-1β (KE10003) and TNF-α (KE10002) (Proteintech, Wuhan, China) were used according to the manufacturer’s protocols. The data are presented as pg of cytokine for mg of the total protein in the samples.

### 4.10. Statistical Analysis

The data were submitted to a 1-way ANOVA followed by post-hoc Sidak, Games–Howell or Tukey’s tests depending on the existence of homoscedasticity for multiple comparisons of the group differences. An assessment of the main factors (age and diet) and their potential interactions was performed by a 2-way ANOVA. Exceptionally, data from the spatial learning quantification in the Barnes maze tests (shown in Figure 1B) were examined by a 3-way ANOVA with a repeated measures test, as three factors (diet, age and the day of training) were combined. The raw data were submitted to pertinent transformations in order to ensure normality. Statistical analyses were performed using the GraphPad (San Diego, CA, USA) or SPSS (IBM, New York, NY, USA) software packages.

## 5. Conclusions

Overall, we proposed that a n-3 LCPUFA-enriched diet induces beneficial cognitive effects that may be partially explained at the molecular level by the reversion of the hippocampal lipid raft microenvironment during aging to the levels of young animals. Consequently, dietary maintenance of these microdomains during aging may stabilize the content of memory-related synaptic markers (NMDA, AMPA and mGlu receptors). ERα integrated in lipid rafts shows similar dynamics in both young and aged female mice exposed to a n-3 LCPUFA-enriched diet. Moreover, the modulation of neuroinflammation by the n-3 LCPUFA-enriched diet could be an additional mechanism implicated in this phenomenon.

We consider that our results may bring new insights into the impact of lipid dietary interventions in hippocampal functioning and neuroinflammation during female aging.

## Figures and Tables

**Table 1:** Fatty composition of lipid rafts isolated from hippocampus.

	6 Months		15 Months	
	CTRL	n-3 LCPUFA	CTRL	n-3 LCPUFA
**C16:0 (Palmitic acid)**	26.91 ± 0.97	27.65 ± 1.93	29.78 ± 1.64	29.99 ± 0.99
**C18:0 (Stearic acid)**	18.45 ± 0.54	19.97 ± 1.44	22.36 ± 1.27	19.31 ± 0.73
**C18:1n9 (Oleic acid)**	14.87 ± 0.51	13.74 ± 0.35	12.45 ± 0.89	13.24 ± 0.75
**C18:2n6 (Linoleic acid)**	1.22 ± 0.36	0.69 ± 0.39	0.81 ± 0.34	0.13 ± 0.13
**C18:3n3 (Linolenic acid)**	0.23 ± 0.10	0.11 ± 0.11	0.14 ± 0.09	0.00 ± 0.00
**C20:4n6 (AA)**	5.85 ± 0.16 ^**a**^	4.97 ± 0.40 ^**ab**^	5.00 ± 0.18 ^**ab**^	4.76 ± 0.24 ^**b**^
**C20:5n3 (EPA)**	1.14 ± 0.44	0.65 ± 0.42	0.76 ± 0.27	0.79 ± 0.27
**C22:5n-3 (DPA)**	0.21 ± 0.07 ^**ab**^	0.36 ± 0.06 ^**a**^	0.14 ± 0.05 ^**b**^	0.42 ± 0.06 ^**a**^
**C22:6n3 (DHA)**	8.64 ± 0.64 ^**a**^	9.22 ± 0.77 ^**a**^	6.33 ± 0.25 ^**b**^	10.10 ± 0.48 ^**a**^
**C24:1n9 (Nervonic Acid)**	1.15 ± 0.25	0.91 ± 0.23	0.77 ± 0.15	1.50 ± 0.41
**Indexes and Totals**				
**n-3 series**	10.17 ± 1.12 ^**a**^	10.45 ± 0.68 ^**a**^	7.33 ± 0.31 ^**b**^	11.40 ± 0.47 ^**a**^
**n-6 series**	8.71 ± 0.66	7.47 ± 0.40	8.08 ± 0.43	6.91 ± 0.27
**Monoenes series**	17.32 ± 0.33 ^**a**^	15.90 ± 0.72 ^**ab**^	14.28 ± 1.08 ^**b**^	16.24 ± 0.63 ^**b**^
**n-6/n-3 ratio**	0.92 ± 0.14 ^**ab**^	0.72 ± 0.02 ^**b**^	1.10 ± 0.05 ^**a**^	0.61 ± 0.03 ^**b**^
**Saturates**	47.87 ± 1.30	49.65 ± 3.04	54.39 ± 2.80	50.99 ± 1.58
**Monoenes**	22.64 ± 0.54	21.02 ± 1.30	19.71 ± 1.39	21.55 ± 0.91
**PUFA**	18.89 ± 0.90 ^**a**^	17.92 ± 1.06 ^**ab**^	15.41 ± 0.67 ^**b**^	18.31 ± 0.61 ^**ab**^
**n-3 LCPUFA**	10.03 ± 1.00 ^**a**^	10.27 ± 0.62 ^**ab**^	7.40 ± 0.22 ^**b**^	11.26 ± 0.48 ^**a**^
**PUFA/Saturates**	0.40 ± 0.02 ^**a**^	0.37 ± 0.03 ^**ab**^	0.29 ± 0.02 ^**b**^	0.36 ± 0.03 ^**ab**^
**UI (Unsaturation Index)**	114.76 ± 5.68 ^**a**^	111.11 ± 4.96 ^**a**^	92.09 ± 2.95 ^**b**^	114.41 ± 3.53 ^**a**^
**PI (Peroxidation Index)**	109.64 ± 7.18 ^**a**^	108.14 ± 6.85 ^**a**^	84.60 ± 3.39 ^**b**^	113.63 ± 4.53 ^**a**^

Results are expressed in mole % and represented as mean ± SEM. Data were submitted to one-way ANOVA followed by Tukey’s test. Values in the same row bearing different letters are significantly different with *p* < 0.05. UI and PI were calculated as described in Díaz et al. (2015) [13] and Cosgrove et al. (1987) [61]. Five animals were used in each group.

**Table 2:** Nutritive content of the standard and n-3 LCPUFA-enriched diets.

Nutritional Additives	Standard Diet (per kg)	n-3 LCPUFA-Enriched Diet (per kg)
Vitamin A	21,000 (UI)	21,000 (UI)
Vitamin D_3_	1100 (UI)	1100 (UI)
Iron	50 mg	50 mg
Magnesium	40 mg	40 mg
Zinc	31 mg	31 mg
Copper	7 mg	7 mg
Iodine	6.2 mg	6.2 mg
**Technological additives**	**Standard diet (per kg)**	**n-3 LCPUFA-enriched diet (per kg)**
Sepiolite	760 mg	760 mg
**Analytical constituents**	**Standard diet (%)**	**n-3 LCPUFA-enriched diet (per kg)**
Moisture	12.00	12.00
Crude protein	14.50	14.50
Crude fibers	4.50	4.50
Crude ash	4.70	4.70
Crude oil and fats	4.00	4.82
**Of which fatty acids**	**Standard diet (%)**	**n-3 LCPUFA-enriched diet (%)**
C16:0 (Palmitic acid)	0.5	0.5
C18:0 (Stearic acid)	0.1	0.1
C18:1n9 (Oleic acid)	0.7	0.7
C18:2n6 (Linoleic acid)	2.0	2.0
C18:3n3 (Linolenic acid)	0.1	0.1
C20:5n3 (EPA)	—	0.56
C22:6n3 (DHA)	—	0.26
**Total fatty acids classes**	**Standard diet (%)**	**n-3 LCPUFA-enriched diet (%)**
Total Saturated	0.6	0.6
Total Monounsaturated	0.7	0.7
Total Polyunsaturated	2.1	2.92

Composition: wheat middlings, wheat maize, maize gluten, calcium carbonate, soybean oil, mineral dicalcium phosphate, corn gluten feed, sodium chloride, magnesium oxide.

## Data Availability

All the data presented in this study are available in the same manuscript and its supplementary material.

## Supplementary Materials

The following supporting information can be downloaded at: https://www.mdpi.com/article/10.3390/ijms23137430/s1.
