# Supplementary material for: Effects of Dietary n-3 LCPUFA Supplementation on the Hippocampus of Aging Female Mice: Impact on Memory, Lipid Raft-Associated Glutamatergic Receptors and Neuroinflammation
Source: Int J Mol Sci. 2022 Jul 4;23(13):7430. doi: 10.3390/ijms23137430 (PMC9267073; doi:10.3390/ijms23137430)

Flotillin-1

6 months

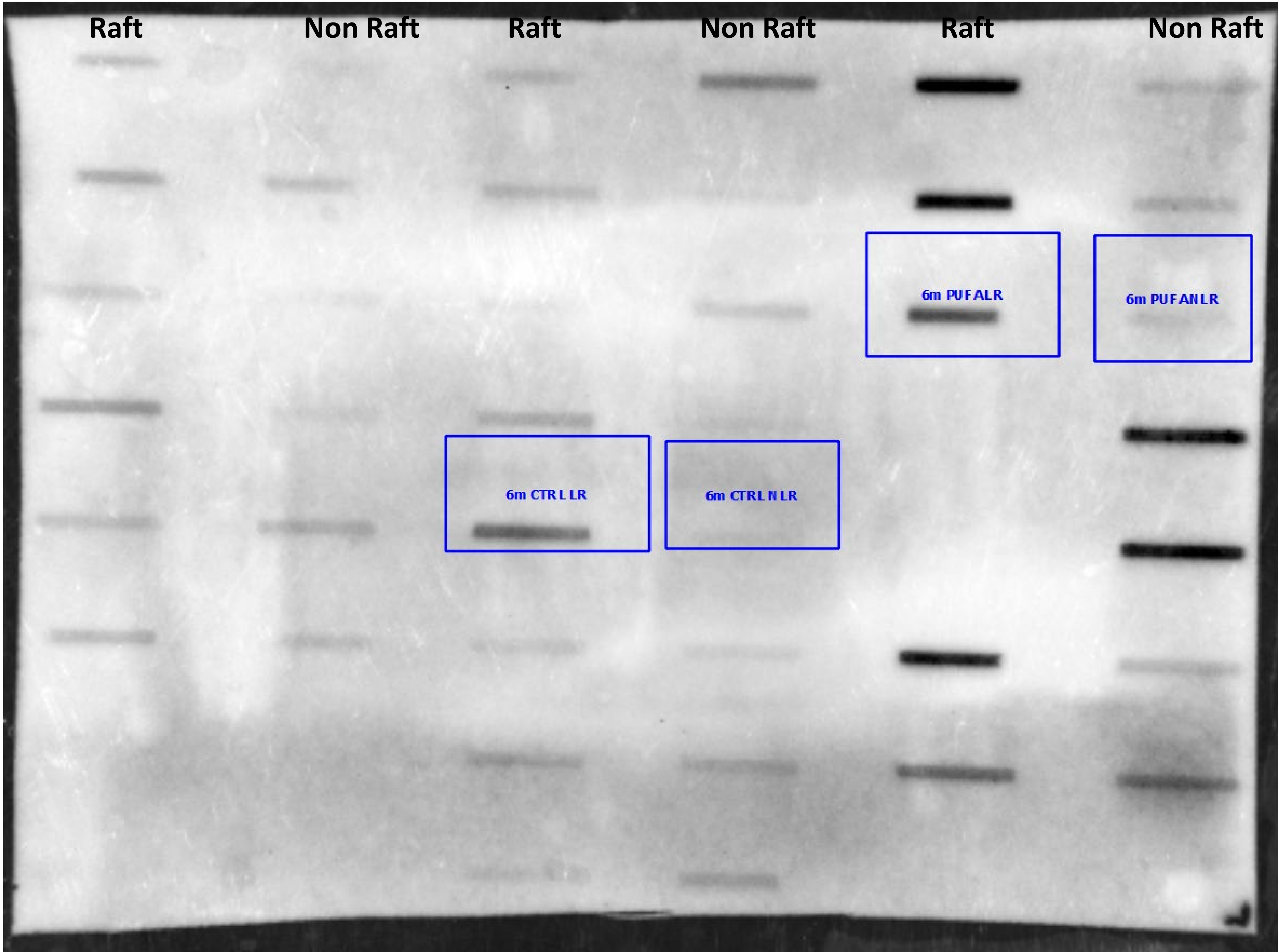

*Nomenclature

LR = Raft
NLR = Non Raft

Flotilin-1

15 months

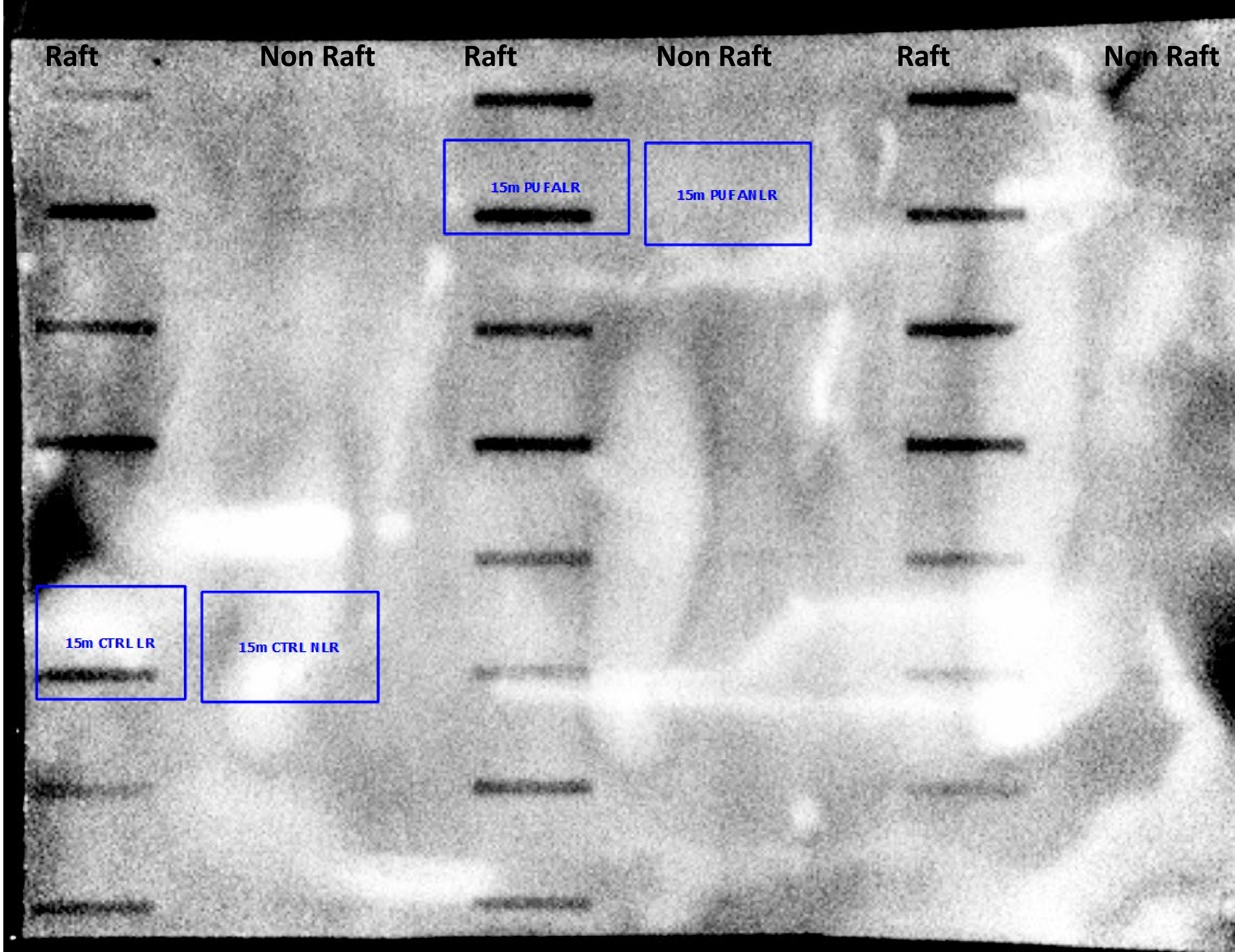

mGluR5

6 and 15 months

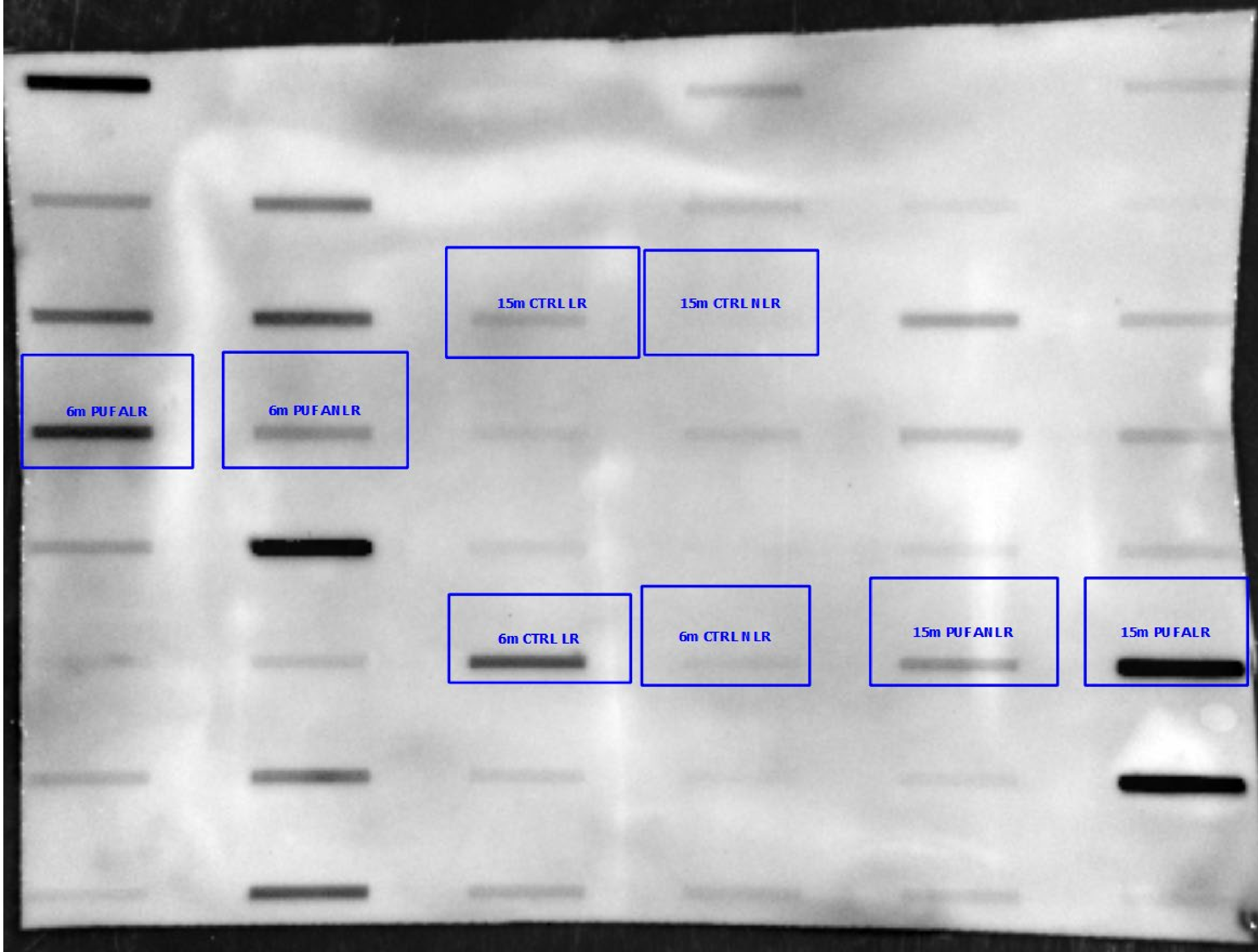

GluN2B

6 and 15 months

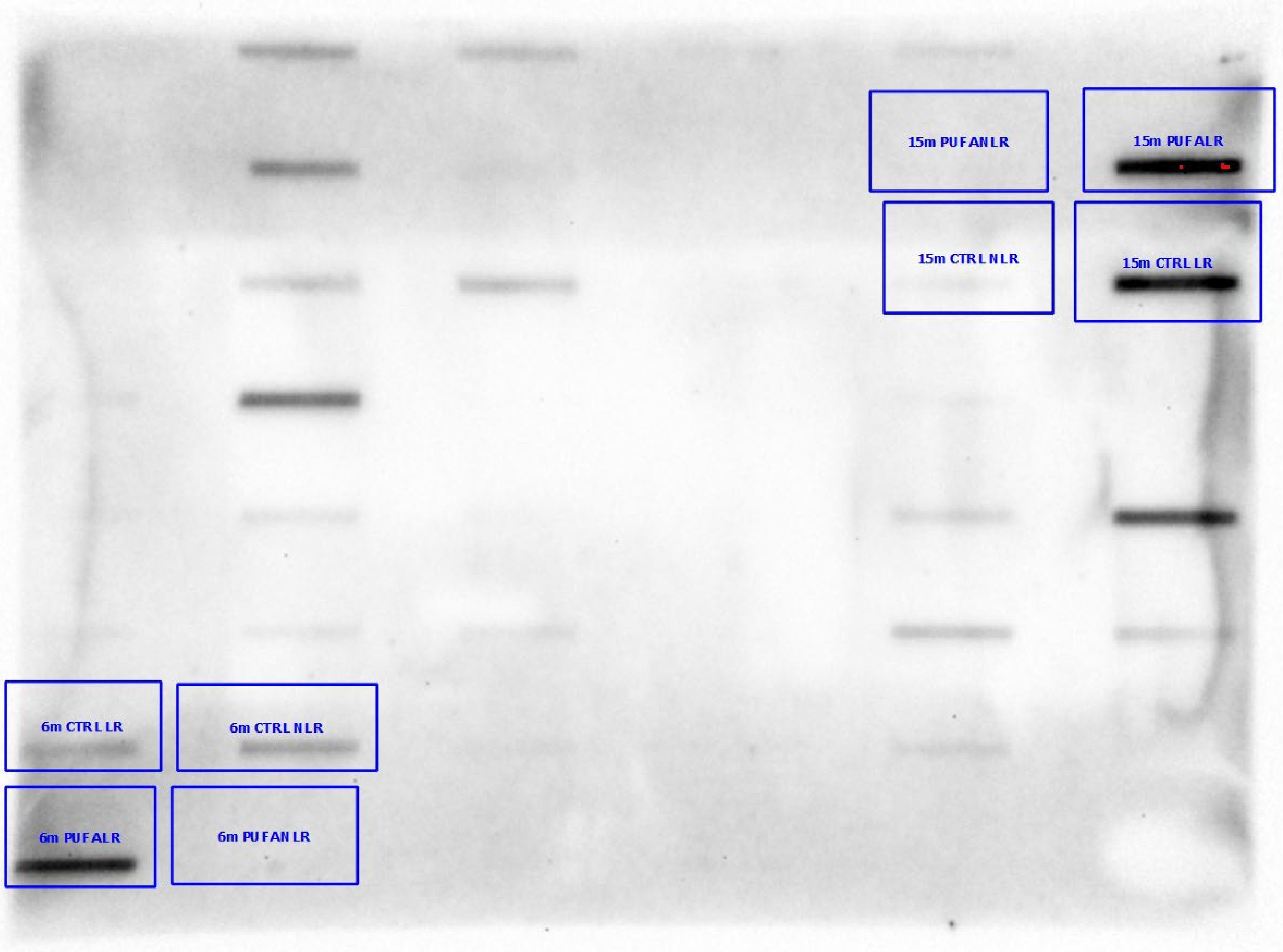

GluA1

6 and 15 months

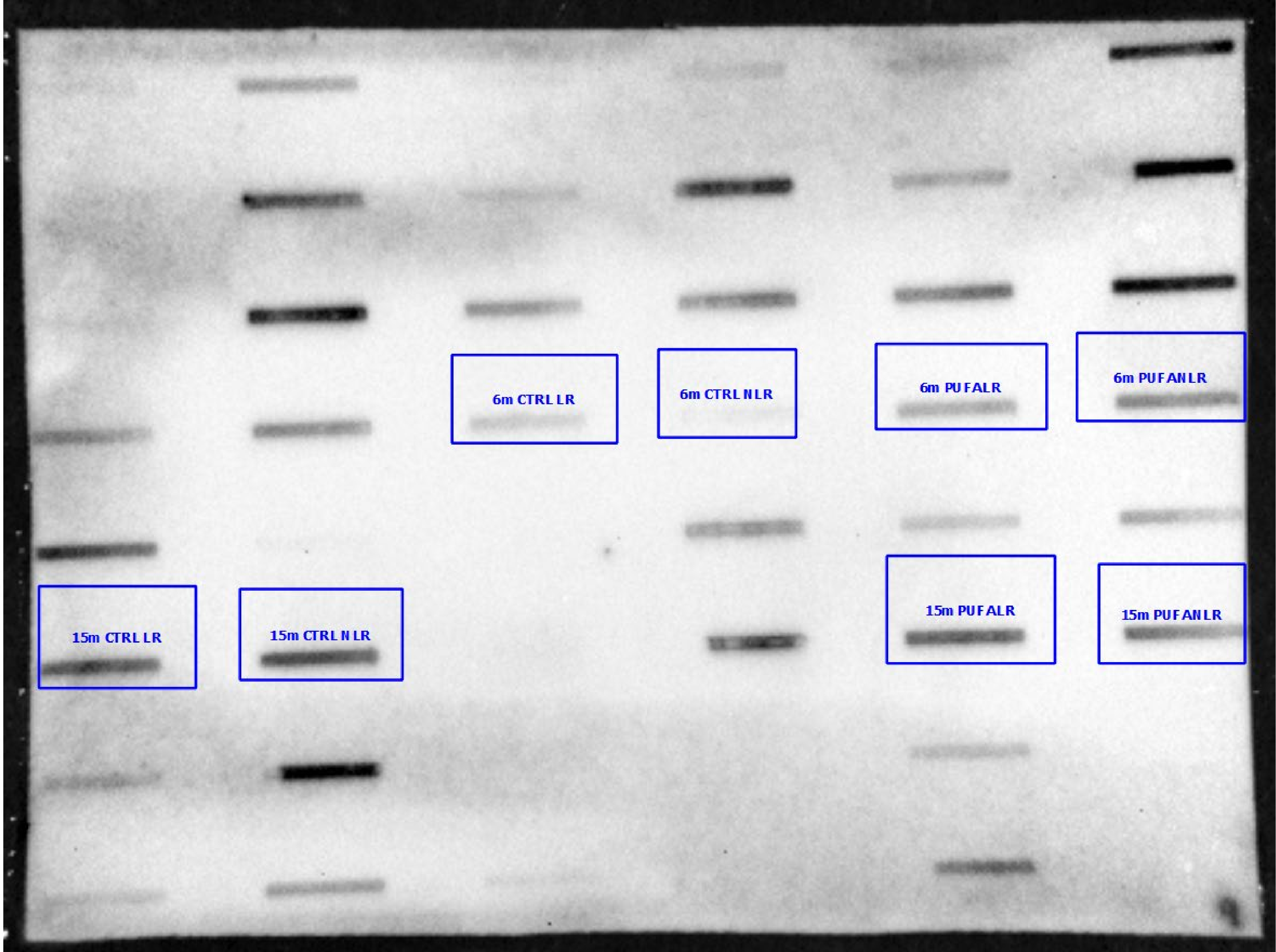

ERalpha

6 and 15 months

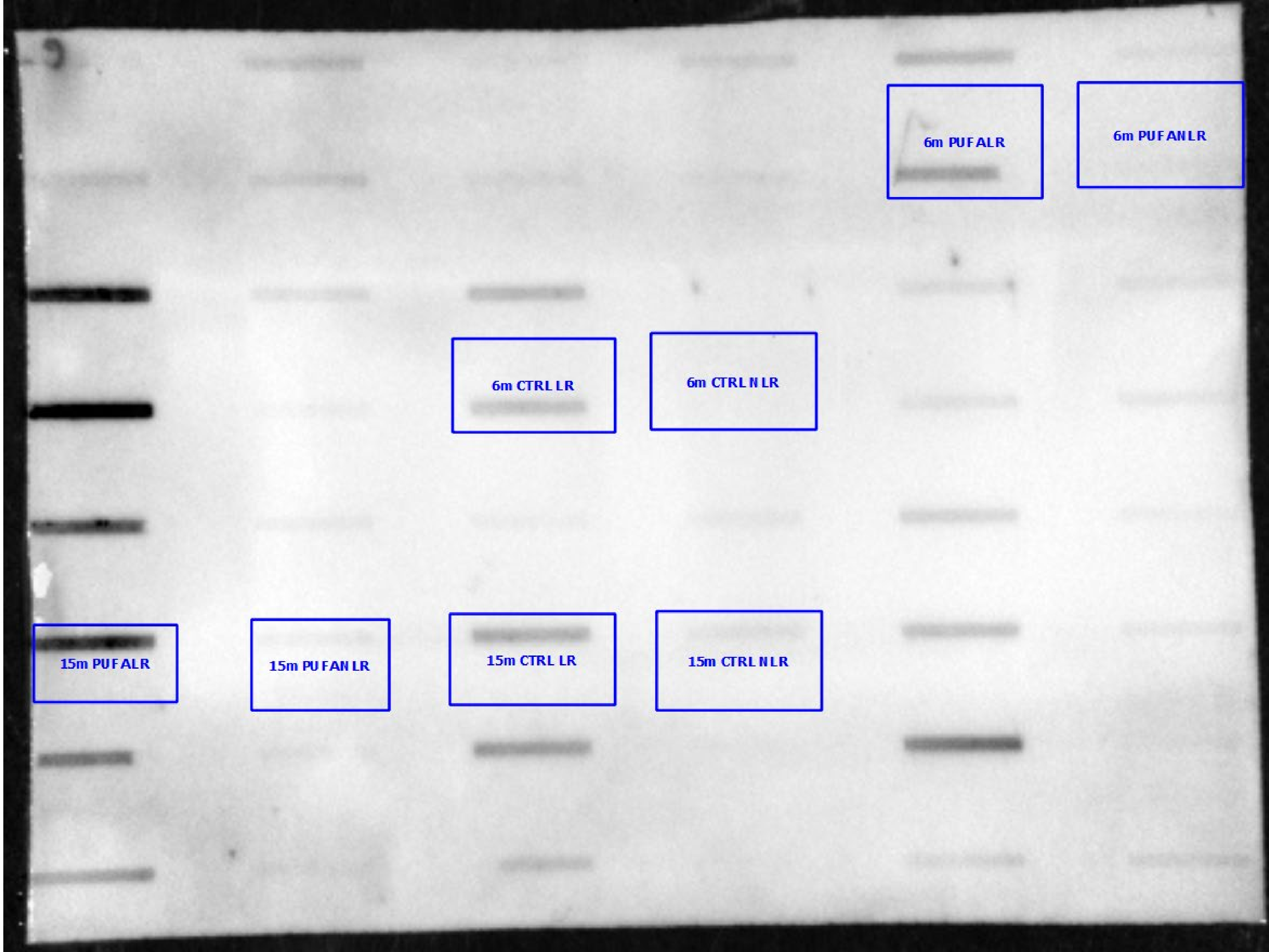

Tubulin

6 and 15 months

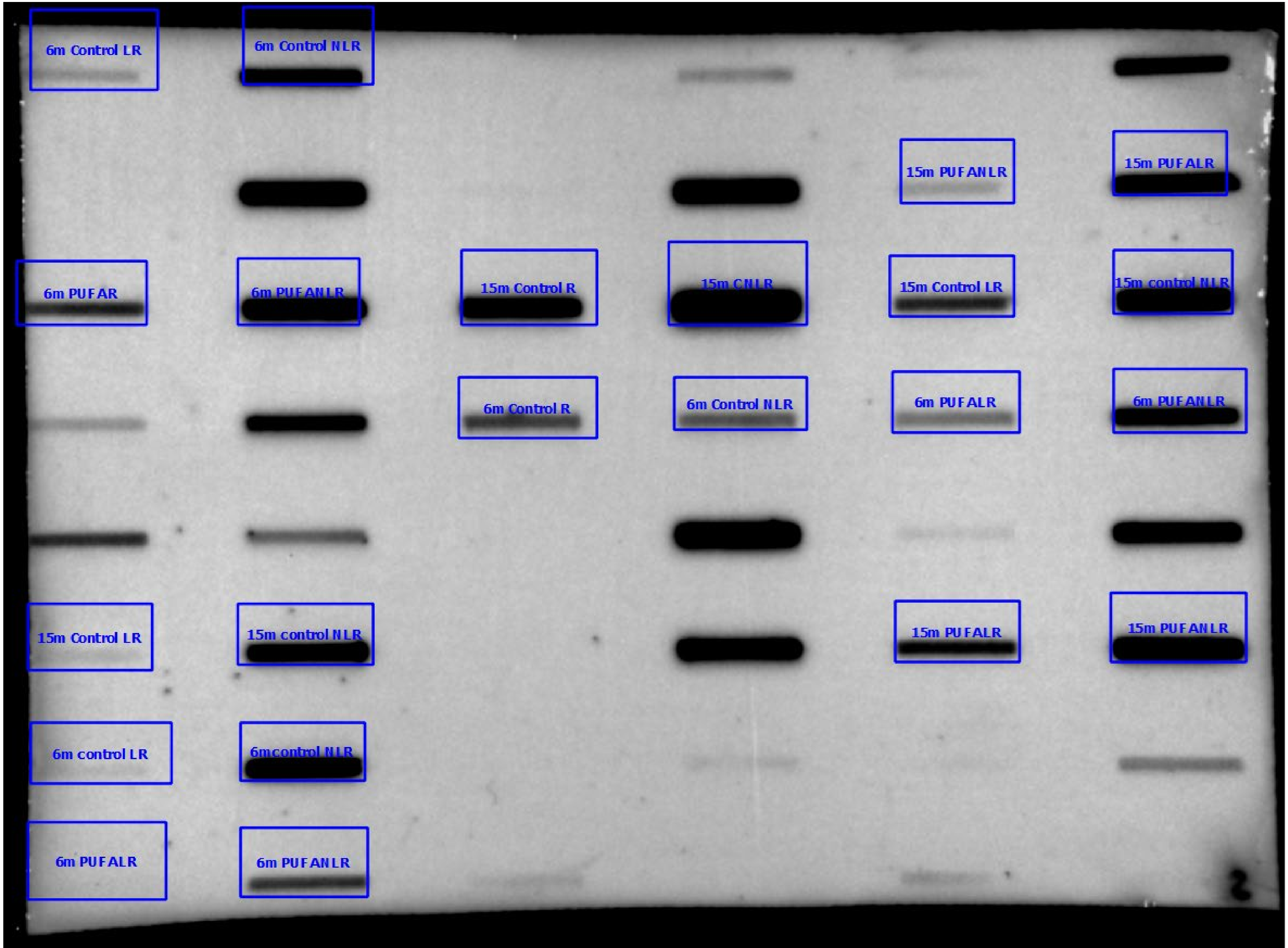

Tubulin

6 and 15 months

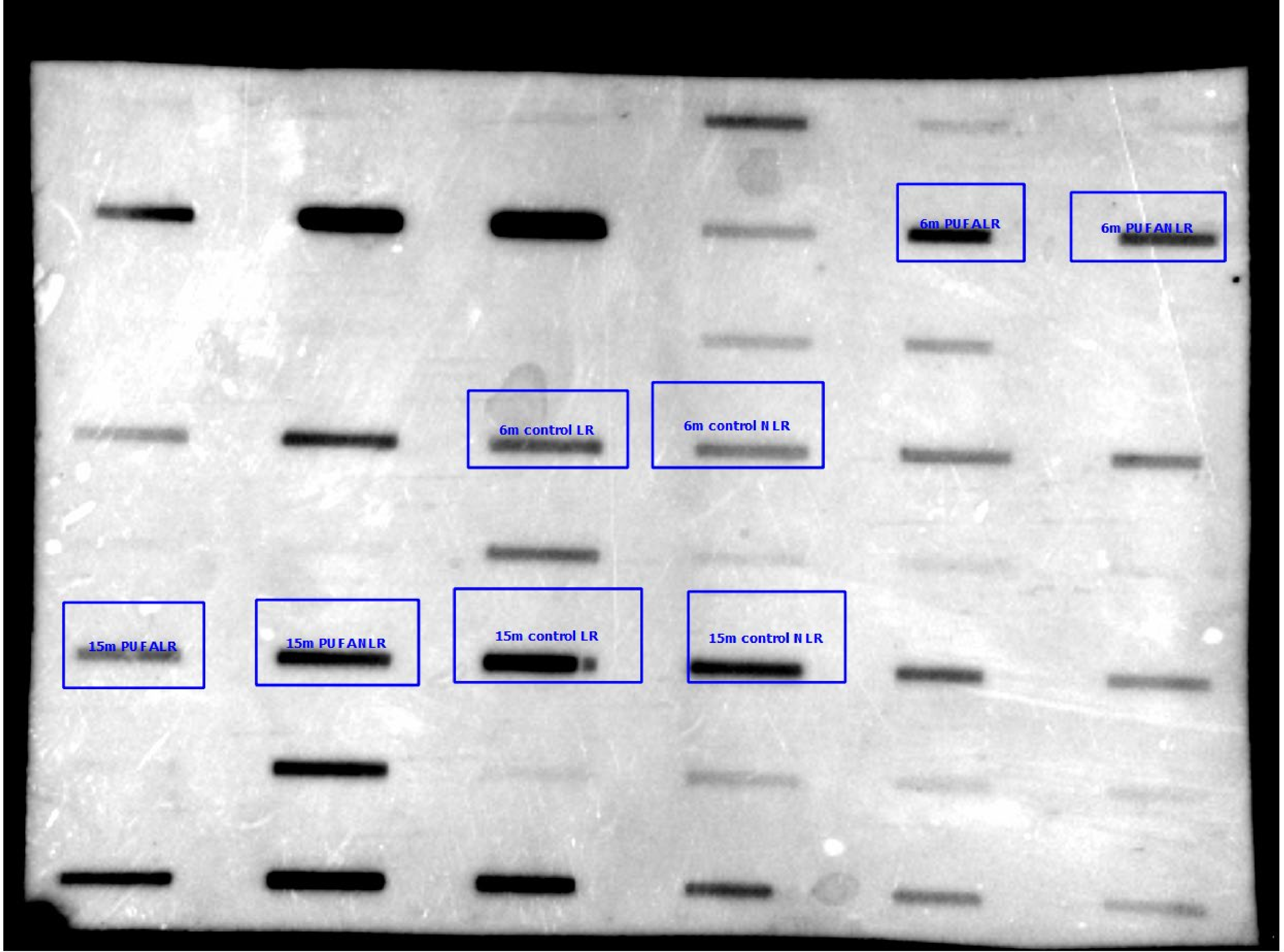

Tubulin

15 months

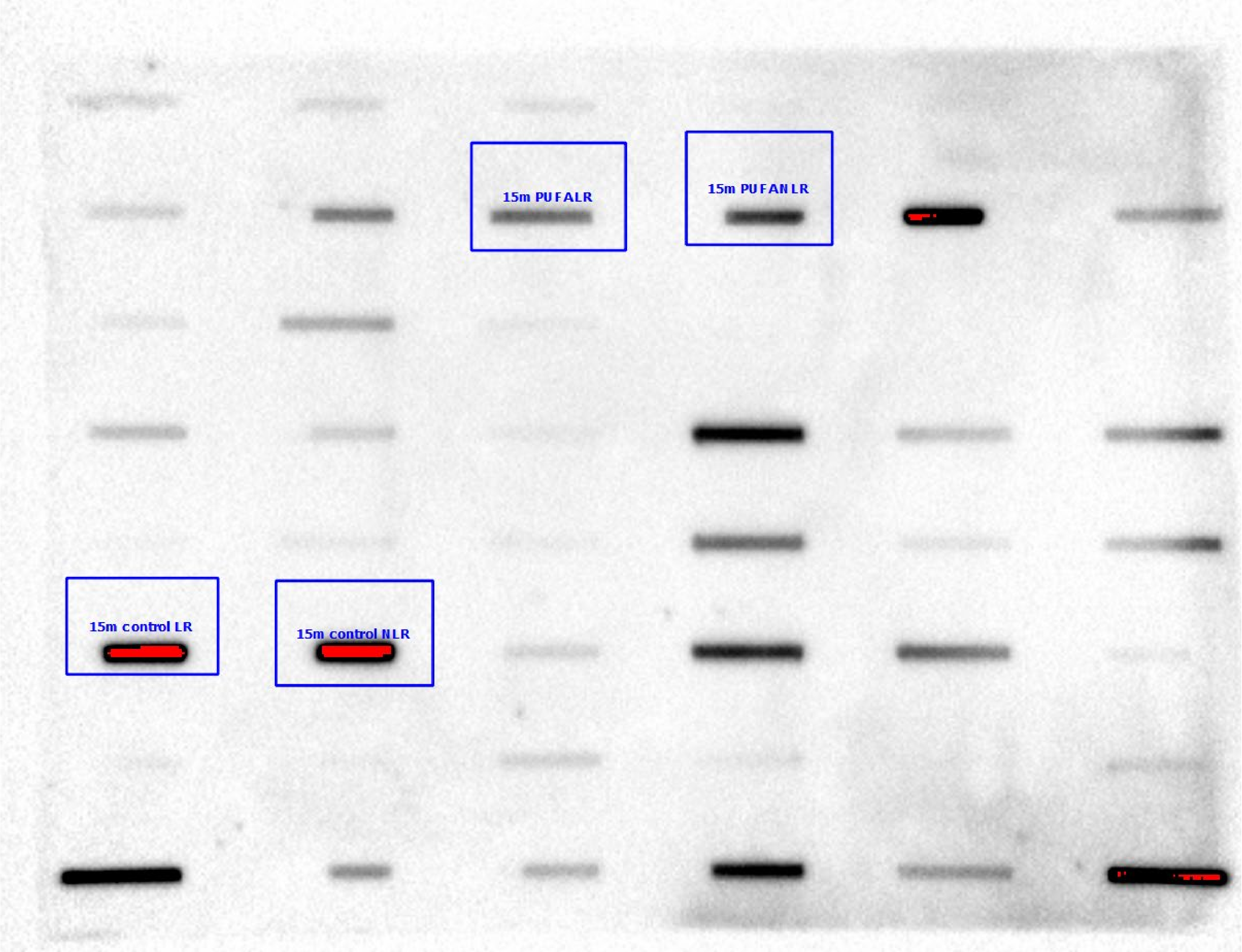

Tubulin

6 months

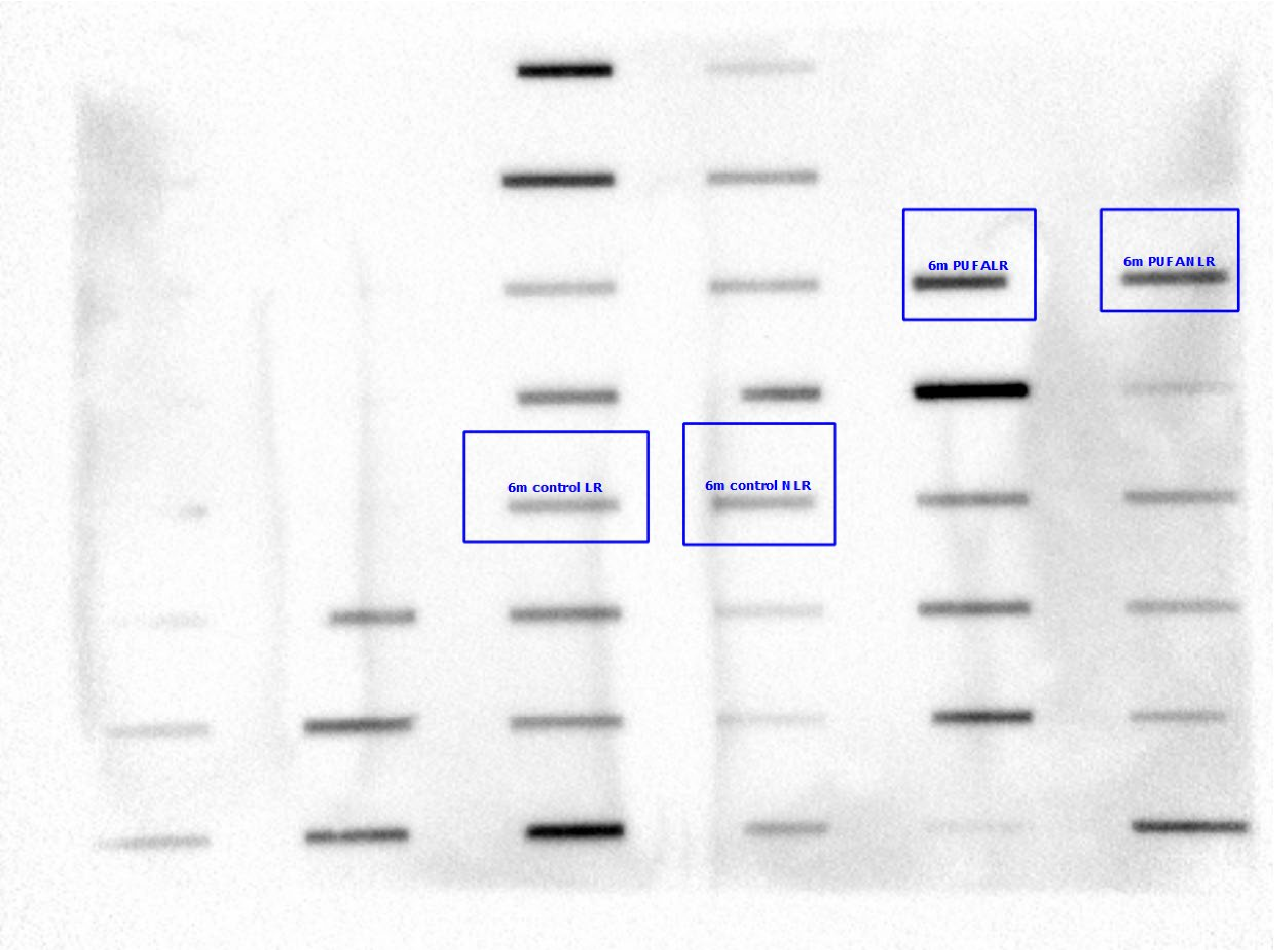

Supplement: Supplementary file 1 [file ijms-23-07430-s001.zip › ijms-1799110-supplementary.pdf]
